# The Cholera and the Law

**Published:** 1892-10

**Authors:** 


BUFFALO MEDICAL AND SURGICAL JOURNAL

A MONTHLY REVIEW OF MEDICINE AND SURGERY.

*EDITORS:*

THOMAS LOTHROP, M. D. - - WM. WARREN POTTER, M. D.

All communications, whether of a literary or business character, should be addressed to the managing editor: 284 Franklin Street, Buffalo, N. Y.

Vol. XXXII. OCTOBER, 1892. No. 3.

### THE CHOLERA AND THE LAW.

It is a matter of great satisfaction, and it ought to be the pride of the citizens of Buffalo, that its health department is manifesting such activity and efficiency in making all reasonable preparations to meet any possible advent of cholera. It is true that the possibility of its reaching Buffalo during this season may be a remote one, but it cannot be denied that it is raging in some parts of Europe with fearful havoc, and that it has even been brought to our very doors, whose thresholds, happily, it has thus far not been allowed to cross. But it will not do to act upon this possibility when steamships are arriving at our chief port of entry, almost daily, with cases of cholera on board.

Dr. Ernest Wende and his able assistants, Drs. Pettit and Heath, together with their several coadjutors in the work, ought to receive the support of every respectable citizen in their attempt to put the city in a thorough sanitary condition. At a time like this it is not pertinent to quarrel over details, or to utter captious criticisms of minor acts. We hesitate not to declare that any individual who does so, either by voice or pen, deserves the anathemas of every decent well-thinking man and woman. It is a fortunate thing that the health authorities are clothed with plenary powers, hence are not obliged to ask the other departments as to how far they may go in the performance of their duties. Unless they proceed to apply the most extreme measures of prevention known to sanitary science, they will be held to a strict account for any and every case of cholera which should gain entrance into this city ; but, on the other hand, they must be given every opportunity to prevent its advent that an intelligent and liberal people can afford. In the main we believe this has thus far been done, and we bespeak for the future an equally ready compliance with all the orders that may be issued by the health commissioner.

It would seem as though some such threatening danger was needed to stimulate a proper sanitary spirit on the part of the administration of municipal governments. This is comparatively a healthy city, yet in places it wreaks with noisome stenches, and in some others the accumulated filth is amazing. Here is an opportunity for a thorough overhauling of every nook and cranny where filth accumulates, and where disease-producing germs may propagate.

In the lower bay of New York, at the present writing, there are twenty-eight quarantined vessels, some of them containing many cases of cholera, and on board a few deaths are occurring daily. The wisdom of the national quarantine circular has not been questioned anywhere, so far as we know, except by the chief quarantine officer at New York, Dr. William T. Jenkins. It was given out that Dr. Jenkins proposed to pay no attention to this order, but would act on his own judgment with reference to the period of quarantine. In the presence of such danger as seems imminent, we have no patience with any quibbling subordinate officer who attempts to assert his own authority and to set up his own rule of action in defiance of the national supremacy. If these conflicts are likely to arise between State and Nation on the subject of quarantine in time of threatened epidemic, then it would appear wise for the United States Government to establish its own station, and maintain it at its own expense, leaving the State of New York to conduct its little affairs in its own way on interior lines.

We repeat that with cholera ships daily arriving in New York bay, there is no time to lose in applying the most stringent preventive measures. There should be no dispute over minor details. The law affords ample protection. Let the people cheerfully obey it as expounded by the officers whose duty it is to execute it. The health officers have no easy task to perform, and their burden should not be made heavier by opposition either from the people or the coördinate branches of the municipal government.

